# Clinical severity of rhinovirus/enterovirus compared to other respiratory viruses in children

**DOI:** 10.1111/irv.12255

**Published:** 2014-05-07

**Authors:** Sandra A Asner, Astrid Petrich, Jemila S Hamid, Dominik Mertz, Susan E Richardson, Marek Smieja

**Affiliations:** a Division of Infectious Diseases, Department of Paediatrics, The Hospital for Sick Children, University of TorontoToronto, ON, Canada; b Division of Microbiology, Department of Paediatric Laboratory Medicine, The Hospital for Sick ChildrenToronto, ON, Canada; c Department of Pathobiology and Laboratory Medicine, University of TorontoToronto, ON, Canada; d Department of Clinical Epidemiology and Biostatistics, McMaster UniversityHamilton, ON, Canada; e Department of Pathology and Molecular Medicine, St Joseph's HealthcareHamilton, ON, Canada; f Department of Medicine, Juravinski Hospital and Cancer CenterHamilton, ON, Canada

**Keywords:** Clinical disease severity, human rhinovirus/enterovirus, influenza, respiratory syncytial virus, single viral infections

## Abstract

**Background:**

Human rhinovirus/enterovirus (HRV/ENT) infections are commonly identified in children with acute respiratory infections (ARIs), but data on their clinical severity remain limited.

**Objectives:**

We compared the clinical severity of HRV/ENT to respiratory syncytial virus (RSV), influenza A/B (FLU), and other common respiratory viruses in children.

**Patients/Methods:**

Retrospective study of children with ARIs and confirmed single positive viral infections on mid-turbinate swabs by molecular assays. Outcome measures included hospital admission and, for inpatients, a composite endpoint consisting of intensive care admission, hospitalization >5 days, oxygen requirements or death.

**Results:**

A total of 116 HRV/ENT, 102 RSV, 99 FLU, and 64 other common respiratory viruses were identified. Children with single HRV/ENT infections presented with significantly higher rates of underlying immunosuppressive conditions compared to those with RSV (37·9% versus 13·6%; *P* < 0·001), FLU (37·9% versus 22%; *P* = 0·018) or any other single viral infection (37·9% versus 22·5%; *P* = 0·024). In multivariable analysis adjusted for underlying conditions and age, children with HRV/ENT infections had increased odds of hospitalization compared to children with RSV infections (OR 2·6; 95% CI 1·4, 4·8; *P* < 0·003) or FLU infections (OR 3·0; 95% CI 1·6, 5·8; <0·001) and increased odds of severe clinical disease among inpatients (OR 3·0; 95% CI 1·6,5·6; *P* = 0·001) when compared to those with FLU infections.

**Conclusions:**

Children with HRV/ENT had a more severe clinical course than those with RSV and FLUA/B infections and often had significant comorbidities. These findings emphasize the importance of considering HRV/ENT infection in children presenting with severe acute respiratory tract infections.

## Introduction

Human rhinovirus/enterovirus (HRV/ENT) has been recently identified as the leading pathogen in acute asthma exacerbations, bronchiolitis, and viral pneumonia, although the clinical severity of respiratory illnesses attributed to HRV/ENT remains uncertain.^1^–^10^ Studies conducted among younger children admitted for bronchiolitis reported conflicting information about the clinical severity of HRV/ENT infections, possibly as a result of different age groups studied.^11^,^12^ A study by Papadopulos *et al*.,^4^ reported that HRV/ENT was a significant predictor for severe disease, another study by Midulla *et al*.^12^ reported that HRV/ENT bronchiolitis resulted in less severe disease compared to RSV bronchiolitis among infants. Conversely, studies involving older hospitalized children^1^,^5^,^13^ consistently reported equivalent disease severity between respiratory illnesses caused by HRV/ENT and those caused by other common respiratory viruses. Explanations for the discrepancies observed among these studies^4^,^12^,^13^ include the use of different clinical criteria to assess clinical severity (clinical severity scores versus clinical outcomes) and the focus on different patient populations and age groups (inpatients and young children). Furthermore, the contribution of HRV/ENT infection versus host-related factors such as underlying immunosuppression to the severity of the disease was not reported in most studies.

In summary, there is growing evidence that HRV/ENT is the most commonly identified virus in LRTIs in both adults and children and thus needs to be regarded as a pathogen responsible for much more than just the common cold. The objective of this study was to compare the clinical characteristics and the severity of illness of HRV-/ENT-positive children to those positive for RSV A/B, FLU-A/ FLU-B, and other respiratory viruses by (i) using a large dataset with a broad pediatric inpatient and outpatient population; (ii) considering several clinical endpoints to assess disease severity, and (iii) conducting multivariable analysis to adjust for potential confounding variables.

## Materials and methods

### Participants and definitions

We conducted a single-center retrospective study of children presenting with an acute respiratory illness and any single viral infection documented by molecular assays from mid-turbinate swabs to the Hospital for Sick Children, Toronto, Canada. Multiplex PCR was conducted in a random sample of all patients presenting with ARTI in whom the treating physician ordered a mid-turbinate swab. Testing for respiratory viruses relied on clinical assessment as routinely done in children with respiratory illnesses. These included common cold, pharyngitis, laryngitis, tracheitis, bronchitis, bronchiolitis, and community-acquired pneumonia (CAP).

We used a dataset from the microbiology laboratory at the Hospital for Sick Children, which originally aimed to compare the sensitivities of four different multiplex assays: ResPlex II v2.0 (Qiagen, Mississauga, ON, Canada); Seeplex RV15 kit (Seegene Inc., Seoul, Korea); xTAG-RVP and xTAG-RVP Fast, (Luminex, Austin, TX, USA) in addition to DFA and viral culture. The results from the original study were recently published elsewhere.^14^ The dataset was augmented through retrospective chart review. Children with documented viral coinfections (i.e., detection of more than one viral pathogen in the same sample) were excluded as their inclusion would not allow single viral infection comparisons as a result of not being able to determine which of the viruses detected was the leading pathogen. Data were collected from November 2007 to April 2008 and January 2009 to March 2009, the times when multiplex PCR testing was routinely utilized in randomly selected patients under 18 years of age presenting with acute respiratory tract infections (ARTIs). URTI was defined as the detection of any single respiratory viral infection together with symptoms involving the upper respiratory tract (nose and pharynx).^15^ LRTI was defined as any patient with cough, tachypnea, and/or any respiratory distress or wheezing. Community-acquired pneumonia (CAP) was defined as any of the above LRTI symptoms with pulmonary infiltrates diagnosed on a chest X-ray by a radiologist. Severe clinical disease was defined by hospital admission for outpatients and by a composite endpoint including intensive care (ICU) admission, hospitalization >5 days, oxygen requirements or death in patients admitted to the hospital. Ethics approval was obtained from the Research Ethics Board at The Hospital for Sick Children.

### Collection of clinical information and specimens

Information on patient demographics, relevant baseline characteristics such as underlying comorbidities and outcomes were all extracted from health records. Relevant underlying comorbidities were grouped into three mutually exclusive categories: cardiorespiratory, prematurity, and any immunosuppressive/metabolic conditions. One or more of the following immunodeficiency states were included in the latter group: hematopoietic stem cell transplant (allogeneic and autologous) and solid organ transplant (SOT) recipients, recipients of cancer chemotherapy or long-term immunosuppression for any chronic disease, and congenital immunodeficiency states. Metabolic conditions included any inherited metabolic diseases such as cystinuria, phenylketonuria (PKU), gout, and thyroid disease. In case of multiple comorbidities, patients were referred to the group considered as the most significant comorbidity: an underlying immunocompromised/metabolic condition was considered most significant comorbidity, followed by a cardiorespiratory comorbidity. Outcomes consisted of hospital admission, duration of hospitalization, admission length >5 days, intensive care (ICU) admission, any supplemental oxygen requirements, presence of URTI, CAP, and all-cause mortality.

### Virology studies

From November 2007 to April 2008 and January 2009 to March 2009, 5345 mid-turbinate swabs (FLOQSwabs, Copan Italia, Brescia, Italy) were tested for respiratory viruses of which 750 (14%) were randomly selected each week among all specimens collected from children with acute respiratory tract infections (ARTIs)) and submitted to the clinical laboratory for routine virology testing including molecular assays. Neither laboratory staff nor clinicians were aware in advance which patient would be selected for viral detection by molecular assays. Swabs were assayed by four different nucleic acid amplification-based assays: ResPlex II v2.0 (Qiagen); Seeplex RV15 kit (Seegene Inc.); xTAG-RVP and xTAG-RVP Fast (Luminex). These assays detect up to 18 respiratory viruses [RSV (A, B), coronaviruses (OC43, 229E, NL63, HKU1), rhinovirus/enterovirus (HRV/ENT), coxsackie/echovirus, PIV (1, 2, 3, 4), FLU-A, FLU-B, bocavirus (HBoV), ADV (A,B,C,D,F). and hMPV]. ResPlex II v2.0, and Seeplex RV15 assays distinguished single HRV infections from single ENT infections whereas xTAG-RVP and xTAG-RVP fast assays reported a combined result of HRV/ENT as reported in our study. These combined results did include coxsackie and echoviruses. All specimens were also examined by direct fluorescent antigen assay (DFA) for eight respiratory viruses, respiratory syncytial virus (RSV), influenza virus [A,B] (FLU-A/FLU-B), parainfluenza [1–3] (PIV), adenovirus (ADV) (SimulFluor®; Millipore, Temecula, CA, USA), and human metapneumovirus (hMPV) (Diagnostic HYBRIDS, Athens, OH, USA) and/or viral culture. We defined a viral result as a true positive if positive by viral culture regardless of other tests; if positive by DFA and at least one molecular test; or if positive by two different molecular tests. For those only detectable by molecular assays (HRV/ENT, coronaviruses, HBoV and PIV 4), a true positive result was defined as two or more positive test results from among the four molecular assays.^14^ Bacterial coinfection was defined as the presence of any bacterial pathogen, identified by culture from blood cultures or respiratory samples (bronchoalveolar lavage, tracheal secretions, sputum samples or pleural fluid) upon initial consultation with respiratory symptoms or within 30 days of their initial consultation. *Staphylococcus epidermidis* positive blood cultures were considered as pathogens if drawn from more than one peripheral blood culture, from one blood culture drawn from a central line, or from one peripheral line in high-risk patients such as those with underlying immunosuppressive conditions, prosthetic devices, or newborns according to recent guidelines.^16^ In addition, a positive urinary antigen for *Streptococcus pneumonia* retrieved upon admission or within 30 days of admission was also considered as bacterial coinfection. Positive bacterial urinary or stool cultures and bacterial pathogen identified from skin or wound swabs were not considered as bacterial coinfections.

### Statistical analyses

Standard descriptive and comparative statistics were performed on data categorized by viral pathogen, where HRV/ENT was used as the reference and compared to RSV, FLU, or category consisting of all other common viruses including PIV 1–4, hMPV, HBoV, ADV, and coronaviruses. The chi-square test or Fisher's exact test was used to compare categorical variables between groups as appropriate. Multivariable logistic regression was used to compare the clinical correlates of clinical disease severity between children infected with HRV/ENT and those infected with either RSV, FLU, or other common single respiratory viruses. We derived medians, used the Mann–Whitney nonparametric method for comparisons of non-normally distributed continuous data, and used a transformation using the natural logarithm (ln) of the outcome variable when performing multivariable linear regression. A two-sided test was performed, and –a *P* value <0·05 was considered to be statistically significant. Data were analyzed using SPSS statistical software (version 20.0, SPSS Inc, Chicago, IL, USA).

## Results

### Patient characteristics

A total of 742 children evaluated for any respiratory illness were screened for respiratory viruses from mid-turbinate swabs by molecular assays during the study period. Of these, 462 (62·3%) children were detected positive for any respiratory virus. Among these 462 patients, 205 (53·8%) were admitted, and among these inpatients, 143 (37·5%) presented with severe disease. A total of 81 (17·5%) had two or more respiratory viruses and were excluded from this analyses. The remaining, 381 (82·5%), tested positive for a single respiratory virus: 116 (30·4%) HRV/ENT, 102 (26·8%) RSV, 99 (26·0%) FLU, and 64 (16·8%) other respiratory viruses. Our rate of overall bacterial coinfection was low (6·4%).

Among inpatients, children with single HRV/ENT infections were significantly older compared to those with single RSV infections (median age 1·9 years versus 0·4 years, *P* < 0·002). Children with HRV/ENT infections presented with a significantly higher rate of underlying cardiorespiratory comorbidities compared to children with RSV (37·6% versus 14·6%; OR 3·5; 95% CI 1·3, 9·3; *P* = 0·01), FLU (37·6% versus 6·5; OR 9; 95% CI 4·0, 38·0; *P* = 0·005), or any other single viral infection (37·6% versus 14·6%; OR 3·3; 95% CI 1·4, 9·0; *P* = 0·007) (Table1).

**Table 1:** Baseline characteristics and clinical outcomes of children admitted in hospital presenting with a single viral acute respiratory tract infection as compared to HRV/ENT

	HRV/ENT*	RSV	FLU	Others
	*n* = 85	*n* = 40	*n* = 46	*n* = 34
Age, years Median, IQR	1·9 (0·4**–**5·8)	0·4 (0·1**–**1·5) ***P*** **< 0·002**	4·0 (1·5**–**9·0) *P* = 0·09	(0·7**–**5·0) *P* = 0·903
Gender, male *N*, %	48 (56·5)	26 (63·4) OR 1·3 95% CI 0·6, 2·9 *P* = 0·459	21 (67·7) *OR 1·6* *95% CI 0·7, 3·9* *P* = 0·276	36 (75) *OR 2·3* *95% CI 1·1, 5·1* ***P*** **= 0·036**
Cardiorespiratory *N*, %	32 (37·6)	6 (14·6) *OR 3·5* *95% CI 1·3, 9·3* ***P*** **= 0·01**	2 (6·5) *OR 9* *95% CI 4·0, 38·0* ***P*** **= 0·005**	7 (14·6) *OR 3·3* *95% CI 1·4, 9·0* ***P*** **= 0·007**
Prematurity *N*, %	8 (9·4)	7 (17·1) *OR 0·7* *95% CI 0·3, 1·7* *P* = 0·458	0	4 (8·3) *OR 0·9* *95% CI 0·3, 3·1* *P* = 0·835
Immunocompromised/metabolic *N*, %	40 (47·1)	13 (31·7) *OR 1·9* *95% CI 0·9, 4·2* *P* = 0·104	12 (38·7) *OR 1·4* *95% CI 0·6, 3·3* *P* = 0·425	16 (33·3) *OR 1·8* *95% CI 0·9, 3·7* *P* = 0·125
Pneumonia *N*, %	28 (32·9)	8 (19·5) *OR 2·4* *95% CI 0·9, 6·9* *P* = 0·093	8 (25·8) *OR 1·9* *95% CI 0·7, 4·9* *P* = 0·191	16 (33·3) *OR 1·3* *95% CI 0·6, 2·7* *P* = 0·609
Length of hospital admission (days) Median, IQR	8 (3**–**19·5)	6 (3**–**9) *P* = 0·429	4 (2**–**11) ***P*** **= 0·035**	8 (3**–**19·5) *P* = 0·943
Admission in the ICU *N*,%	25 (29·4)	14 (34·1) *OR 1·2* *95% CI 0·6, 2·8* *P* = 0·590	6 (19·4) *OR 0·7* *95% CI 0·6, 4·7* *P* = 0·282	15 (31·3) *OR 1·1* *95% CI 0·5, 2·4* *P* = 0·824
Oxygen requirements *N*, %	40 (47·1)	28 (68·3) *OR 2·4* *95% CI 1·1, 5·3* ***P*** **= 0·027**	11 (35·5) *OR 1·6* *95% CI 0·7, 3·8* *P* = 0·268	22 (45·8) *OR 1·1* *95% CI 0·5, 2·1* *P* = 0·892
Mortality** *N*, %	4 (4·7)	0	1 (3·2)	2 (4·2)

HRV/ENT *, enterovirus/rhinovirus: risk estimates, 95% CI and *P* values are indicated in each row for the comparison with HRV/ENT for each categorical variable; RSV, respiratory syncytial virus; FLU, influenza; others: PIV, parainfluenza; hMPV, human meta-pneumovirus; ADV, adenovirus, coronaviruses, HBoV; IQR, interquartile range.

** No risk estimates provided given the small number of events.

Values indicated in bold were considered as significant P < 0.05.

### Clinical outcomes

Children with HRV/ENT infections had significantly higher rates of pneumonia compared to those with RSV (24·1% versus 9·7%; OR 2·3; 95% CI 1·1–5·3; *P* = 0·044). When compared to children with RSV infections or FLU infections, those with HRV/ENT infections had significantly higher admission rates (73·3% versus, respectively, 39·8% and 37·8%; both *P* < 0·001). Among children who were admitted to hospital, those with HRV/ENT infections had an increased length of stay compared to those with FLU infections (8 days, IQR 3–19·5 versus 4 days; IQR 2–11 days; *P* = 0·035). Of six children who died, four presented with HRV/ENT infections; one presented with a progressive acute respiratory distress syndrome (ARDS) after ADV infection and died 4 weeks after FLU-A infection; and the remaining one presented with an HBoV infection. Of the four fatalities with HRV/ENT infections, three presented with underlying immunosuppressive conditions and one with a cardiac comorbidity. Two of the patients died from a sepsis-like picture with respiratory failure and possible underlying pneumonia with no other pathogens identified, thus suggesting that HRV/ENT may have potentially contributed to the mortality. The remaining two patients likely died of their underlying disease (Table1).

### Predictors of hospitalization

In multivariable analysis adjusted for age and comorbidities, we identified that children with HRV/ENT infections were significantly more likely to be hospitalized compared to those with RSV infections (OR 2·6; 95% CI 1·4, 4·8; *P* < 0·003) or FLU infections (OR 3·0; 95% CI 1·6–5·8; <0·001) (Tables2). Furthermore, age (OR 1·1 per year; 95% CI 1·0, 1·2; *P* = 0·009) and underlying immunosuppressive/metabolic conditions (OR 6·5; 95% CI 3·4, 12·5; *P* < 0·001) were significant predictors of hospital admission. An interaction term measuring the combination of HRV-/ENT-positive status and underlying immunocompromised condition was not significant, and its inclusion did not affect the risk estimates above. *Post hoc* analyzes conducted among patients with an underlying immunosuppressive condition did not affect the risk estimates above although our results were not statistically significant as a result of a significantly lower sample size.

**Table 2:** Predictors of hospitalization

	Univariable analysis			Multivariable analysis		
		
	OR	95% CI	*P* value	OR	95% CI	*P* value
HRV/ENT versus RSV	4·3	2·4, 7·5	**<0·001**	2·6	1·4, 4·8	**0·003**
HRV/ENT versus FLU	3·2	1·8, 5·6	**<0·001**	3·0	1·6, 5·8	**0·001**
HRV/ENT versus others	2·4	1·3, 4·6	**0·007**	1·7	0·8, 3·5	0·119
Age	1·1	1·1, 1·2	**<0·001**	1·1	1·0, 1·2	**0·009**
Cardiorespiratory	2·0	1·1, 3·4	**0·014**	1·5	0·8, 2·7	0·223
Prematurity	1·7	0·8, 3·8	0·192	**–**	**–**	**–**
Immunocompromised Metabolic	8·2	4·4, 15·4	**<0·001**	6·5	3·4, 12·5	**<0·001**

HRV/ENT, enterovirus/rhinovirus; RSV, respiratory syncytial virus; FLU, influenza; others: PIV, parainfluenza; hMPV, human meta-pneumovirus; ADV, adenovirus, coronaviruses, HBoV.

Values indicated in bold were considered as significant P < 0.05.

### Predictors of clinical disease severity and admission length among inpatients

Human rhinovirus-/enterovirus-infected children were significantly more likely to present with severe clinical disease in multivariable analysis compared with children admitted to hospital with FLU infections (OR 3·0; 95% CI 1·6, 5·6; *P* = 0·001) (Tables3).

Children admitted with HRV/ENT infections had an 80% increase in the length of stay compared to those admitted with FLU infections (β coefficient 0·6, 95% CI 0·1, 1·0; *P* = 0·012) in multivariable analysis, where the logarithm of admission length was used as an outcome variable. Children with underlying immunosuppressive or metabolic states had a 100% increase in the length of admission compared to those without underlying immunosuppressive or metabolic conditions (β coefficient 0·7; 95% CI 0·3, 1·0; *P* < 0·001) (Table4).

**Table 3:** Predictors of severity as measured by a composite endpoint of admission to the intensive care unit, hospitalization >5 days, oxygen requirements, or death

	Univariable analysis			Multivariable analysis		
		
	OR	95% CI	*P* value	OR	95% CI	*P* value
HRV/ENT versus RSV	2·9	1·7, 5·1	**<0·001**	1·7	0·9, 3·2	0·082
HRV/ENT versus FLU	3·6	2·0, 6·4	**<0·001**	3·0	1·6, 5·6	**0·001**
HRV/ENT versus others	2·6	1·4, 4·9	**0·003**	1·8	0·9, 3·6	0·094
Age	1·1	1·0, 1·1	**0·005**	1·0	1·0, 1·1	0·180
Cardio-respiratory	2·7	1·6, 4·6	**<0·001**	2·2	1·2, 4·0	**0·007**
Prematurity	1·2	0·6, 2·6	0·657	**–**	**–**	**–**
Immunocompromised Metabolic	5·3	3·2, 8·7	**<0·001**	4·7	2·8, 8·0	**<0·001**

HRV/ENT, enterovirus/rhinovirus; RSV, respiratory syncytial virus; FLU, influenza; others: PIV, parainfluenza; hMPV, human meta-pneumovirus; ADV, adenovirus, coronaviruses, HBoV.

Values indicated in bold were considered as significant P < 0.05.

**Table 4:** Predictors of admission length (ln transformation) in multivariable analysis adjusted for viral pathogen, age, and underlying comorbidities

	β coefficients	95% CI	*P* value
HRV/ENT versus RSV	0·4	0·06, 0·9	0·085
HRV versus FLU	0·6	0·1, 1·0	**0·012**
HRV versus others	0·07	−0·4, 0·5	0·781
Age	0·008	−0·03, 0·05	0·687
Cardiorespiratory	0·3	−0·1, 0·7	0·160
Prematurity	0·6	0·005, 1·1	0·052
Immunocompromised Metabolic	0·7	0·3, 1·0	**<0·001**

HRV/ENT, enterovirus/rhinovirus; RSV, respiratory syncytial virus; FLU, influenza; others: PIV, parainfluenza; hMPV, human meta-pneumovirus; ADV, adenovirus, coronaviruses, HBoV.

Values indicated in bold were considered as significant P < 0.05.

## Discussion

Three important observations made in our study were that HRV/ENT acute respiratory infections were very commonly detected in our patient population; children with HRV/ENT infections had more severe outcomes compared to those with other common respiratory viruses; and children with underlying cardiorespiratory or immunocompromised/metabolic conditions were more likely to present with HRV/ENT infections.

The proportion of single HRV/ENT infections reported in our study (30·4%) was significantly higher than in studies which used conventional diagnostic methods (5%),^6^,^17^–^19^ but comparable to those in which molecular tests were used, including studies of children with ARTIs (27·2%)^2^,^11^ and infants with bronchiolitis (29%).^4^ These discrepancies can be attributed to the higher sensitivity and broader range of virus detection by molecular-based methods.

Similar to others studies,^2^,^4^,^5^,^20^ children infected by HRV/ENT alone were younger compared to those with FLU infections but older compared to those with RSV infections. In our study, children with HRV/ENT infections presented with higher rates of underlying cardiorespiratory and immunosuppressive conditions compared to those with any other single viral infection. Our findings are similar to those described in adults admitted with HRV/ENT respiratory infections, in whom high rates of underlying immunosuppressive conditions were identified, thus suggesting that these patients may be at higher risk of HRV/ENT infections.^7^,^21^–^23^ The extent to which severity of respiratory illness is attributed to HRV/ENT or to other underlying conditions is yet unclear.

Notwithstanding the high proportion of underlying comorbidities identified among our HRV/ENT population, HRV-/ENT-positive status was identified as a significant independent predictor for hospital admission and resulted in an increased clinical disease severity among inpatients compared with either RSV or FLU, while controlling for underlying comorbidities and age. Furthermore, we found significantly longer duration of hospitalization among children admitted with HRV/ENT infections compared to those admitted with FLU infections. Also, higher rates of pneumonia were observed among children with single HRV/ENT infections compared to those with RSV single viral infections as suggested by a previous study by Malcolm *et al*.^7^ Finally, half of the deaths observed among the four HRV-/ENT-infected children with underlying conditions may have resulted from their HRV/ENT respiratory illnesses as no other viral, bacterial, or fungal pathogens were documented. All these findings contrast with recently published studies^1^,^5^,^13^ which reported equivalent disease severity between subjects with HRV/ENT infections and those with other common viral infections. These studies, however, had major limitations, which may have resulted in not detecting a true difference. Firstly, the studies were conducted in inpatients only. One can speculate that hospitalized patients share a level of comorbidity leading to more similar outcomes regardless of the virus involved.^24^ Furthermore, most of these studies compared HRV/ENT infections to all other viruses combined and did not adjust for underlying comorbidities. While treatment of FLU-A-positive children with oseltamivir might have been expected to improve their prognosis, no children included in this study received oseltamivir, as specimens were collected before the implementation of guidelines advocating for oseltamivir treatment of high-risk children^25^ Thus, the difference in outcomes between HRV/ENT and treated FLU-A may be even more pronounced than found in our study.

An important strength of our study was the inclusion of outpatients, thus enabling the use of hospital admission as a measure of clinical severity. Furthermore, the adjustment for underlying comorbidities in multivariable analysis reinforced the association between HRV/ENT and clinical outcomes. Given our low rate of bacterial coinfections, no adjustment for this variable was made in multivariable analyses. Finally, we had adequate sample size to compare HRV/ENT to a number of different viruses. Potential limitations of our study relate to its retrospective design. However, we believe that most of our patient-related important outcomes could be well assessed though chart review. Similarly, the observational nature of our study may have led to selection bias as not all consecutive patients were tested for respiratory viruses as a result of limited resources. Random sampling ensured an unbiased selection of samples tested by molecular assays. Furthermore, the criteria for using virologic diagnostics as part of routine patient care may have varied during the study. For instance, viral culture was only routinely used from November 2007 to April 2008, which may have affected the comparisons of illness severity between different viruses. Third, we assessed the presence or absence of viruses, but did not measure their viral load; higher viral loads in acutely ill subjects compared with asymptomatic children might have further strengthened the association between HRV/ENT and severe disease as rhinoviruses are also commonly identified in community-based controls.^6^,^26^ Future research should measure viral loads and use sequencing for genotyping analysis to differentiate HRV from enterovirus and to speciate HRV. Recent studies suggest that human rhinovirus species A and C (HRV-A and HRV-C) may be associated with greater clinical severity compared with HRV-B species.^27^,^25^,^28^ Finally, our estimation of bacterial coinfection may have been underestimated as adequate respiratory samples such as BAL are rarely performed in children and pneumonia rarely result in positive blood cultures. Also, URT samples are not necessarily representative of LRT disease. This limitation inherent to studies assessing the severity of respiratory illnesses in children may be overcome in future prospective studies, conducted among children with specific underlying comorbidities (e.g., immunocompromised children), which would include validated molecular assays for detection of both viral and bacterial pathogens from URT samples in all children with ARTI.

In conclusion, our findings provide new insight into the burden and severity of HRV/ENT infections and reinforce the need for routine diagnosis in hospital settings. HRV/ENT infections were very common and associated with more severe disease than other common viruses such as FLU or RSV highlighting the need for development and testing of specific antiviral drugs.
